# Hempseed (*Cannabis sativa*) Peptide H3 (IGFLIIWV) Exerts Cholesterol-Lowering Effects in Human Hepatic Cell Line

**DOI:** 10.3390/nu14091804

**Published:** 2022-04-26

**Authors:** Jianqiang Li, Carlotta Bollati, Martina Bartolomei, Angelica Mazzolari, Anna Arnoldi, Giulio Vistoli, Carmen Lammi

**Affiliations:** Department of Pharmaceutical Sciences, University of Milan, Via Mangiagalli 25, 20133 Milan, Italy; jianqiang.li@unimi.it (J.L.); carlotta.bollati@unimi.it (C.B.); martina.bartolomei@unimi.it (M.B.); angelica.mazzolari@unimi.it (A.M.); anna.arnoldi@unimi.it (A.A.); giulio.vistoli@unimi.it (G.V.)

**Keywords:** hempseed protein, LDLR, SREBP-2, cholesterol metabolism, PCSK9

## Abstract

Hempseed (*Cannabis sativa*) protein is an important source of bioactive peptides. H3 (IGFLIIWV), a transepithelial transported intestinal peptide obtained from the hydrolysis of hempseed protein with pepsin, carries out antioxidant and anti-inflammatory activities in HepG2 cells. In this study, the main aim was to assess its hypocholesterolemic effects at a cellular level and the mechanisms behind this health-promoting activity. The results showed that peptide H3 inhibited the 3-hydroxy-3-methylglutaryl co-enzyme A reductase (HMGCoAR) activity in vitro in a dose-dependent manner with an IC_50_ value of 59 μM. Furthermore, the activation of the sterol regulatory element binding proteins (SREBP)-2 transcription factor, followed by the increase of low-density lipoprotein (LDL) receptor (LDLR) protein levels, was observed in human hepatic HepG2 cells treated with peptide H3 at 25 µM. Meanwhile, peptide H3 regulated the intracellular HMGCoAR activity through the increase of its phosphorylation by the activation of AMP-activated protein kinase (AMPK)-pathways. Consequently, the augmentation of the LDLR localized on the cellular membranes led to the improved ability of HepG2 cells to uptake extracellular LDL with a positive effect on cholesterol levels. Unlike the complete hempseed hydrolysate (HP), peptide H3 can reduce the proprotein convertase subtilisin/kexin 9 (PCSK9) protein levels and its secretion in the extracellular environment via the decrease of hepatic nuclear factor 1-α (HNF1-α). Considering all these evidences, H3 may represent a new bioactive peptide to be used for the development of dietary supplements and/or peptidomimetics for cardiovascular disease (CVD) prevention.

## 1. Introduction

Low-density lipoprotein (LDL) receptor (LDLR) is a cell membrane glycoprotein that functions in the binding and internalizing of circulating cholesterol-containing lipoprotein particles to maintain cholesterol homeostasis in lipoprotein and lipid metabolism [1]. Defects in LDLR function or expression trigger an elevated LDL cholesterol and result in cardiovascular disease (CVD), one of the largest causes of mortality worldwide [2]. The LDLR expression is not only modulated by intracellular cholesterol content, but also regulated by a transcription factor, named sterol-responsive element binding protein-2 (SREBP-2), which plays a pivotal role in LDLR mRNA expression [3]. As another SREBP-2 gene target, 3-hydroxy-3-methylglutaryl coenzyme A reductase (HMGCoAR) is a key factor in intracellular cholesterol biosynthesis. HMGCoAR is the rate-controlling enzyme in the mevalonate pathway and is also regulated by the AMP-activated protein kinase (AMPK) pathway [4]. In more detail, there are three isoforms of sterol regulatory element-binding proteins (SREBPs), including SREBP-1a, –1c, and –2, each having different roles in lipid synthesis. Particularly, SREBP-2 is specific to cholesterol synthesis and is responsible for the LDLR and HMGCoAR transcription. Upon sterol deficiency, the complex of SREBP-2 and SREBP cleavage-activating protein (SCAP) membrane experiences a successive two-step cleavage process in the golgi to liberate the amino-terminal portion of SREBP-2. Subsequently, this portion of SREBP-2 enters the nucleus, followed by the activation of the transcription of LDLR and HMGCoAR by binding to sterol regulatory elements (SREs) [5,6]. The increase of LDLR determines an enhanced clearance of plasmatic LDL-cholesterol with an improvement of dyslipidemia [7].

In addition, proprotein convertase subtilisin/kexin type 9 (PCSK9) is the major regulator of the LDL, which subsequently affects its ability to efficiently remove LDL-cholesterol from circulation. Briefly, PCSK9 binds to LDLR and causes their destruction within lysosomes, whereas its inhibition results in the recycling of LDLR, leading to the internalization of more LDL-cholesterol and a reduction in the blood levels of LDL. Recently, some papers have shown that specific peptides deriving from food and/or plant proteins are efficiently able to lower LDL and are also safe [8], a fact that has attracted the attention of more and more researchers.

Industrial hemp, the non-drug variety of *Cannabis sativa*, has been used for food and fiber for centuries [9,10]. Recently, hempseed protein has become an important source of bioactive peptides, because of its high nutritive potential and purported health benefits. Briefly, whole hempseeds contain 20% to 25% protein [the main components being globulin (60% to 80%) and albumin] that exerts a positive effect in the regulation of organ function and human metabolism [9,11]. This fact has stimulated a great interest especially for research on multifunctional bioactive peptides.

In some of our preceding publications [11,12], hydrolysates derived from hempseed have been shown to provide a hypocholesterolemic effect by dropping the activity of HMGCoAR, which in turn leads to the activation of the LDLR, followed by the improvement of the hepatic cells’ ability to absorb extracellular LDL. Although an increase of the PCSK9 protein levels was detected, the hempseed hydrolysate (HP) showed a hypocholesterolemic effect similar to that of statins. Considering that proteins are hydrolyzed during digestion, the activity may be attributed to specific peptides encrypted in the protein sequences that are released by digestion and absorbed at an intestinal level [13]. Indeed, further experiments on the intestinal trans-epithelial transport revealed that some peptides in the HP were able to pass through the mature Caco-2 cell barrier. Particularly, H3 (IGFLIIWV) is one of these peptides that provides an antioxidant activity in HepG2 cells by modulating the Nrf-2 and iNOS pathways [14], leading to the decrease of cellular H_2_O_2_-induced ROS, NO, and lipid peroxidation levels [15]. Considering the link between inflammation and oxidative stress, the evaluation of the anti-inflammatory effect of H3 was also carried out in HepG2 cells. As expected, H3 modulates the production of pro-inflammatory cytokines (IFN-γ, TNF and IL-6), anti-inflammatory cytokines (IL-10), and NO through the regulation of the NF-κB and iNOS pathways [16], exerting an effective anti-inflammatory capacity [17].

In light of these observations, a deeper mechanistic investigation was undertaken with the following objectives: (1) to assess the inhibitory activity of peptide H3 on HMGCoAR in vitro and in silico; (2) to figure out how peptide H3 may modulate the activity of the key targets involved in cholesterol metabolism, i.e., LDLR, SREBP-2, HMGCoAR, p-AMPK, HNF1-α, and PCSK9; and (3) to evaluate the capacity of HepG2 cells treated with peptide H3 to absorb extracellular LDL cholesterol.

## 2. Materials and Methods

### 2.1. Chemicals

All reagents and chemicals used are commercially available. Specific details are available in the Supplementary Materials.

### 2.2. HepG2 Cell Culture Conditions and Treatment

The HepG2 cell line was bought from ATCC (HB-8065, ATCC from LGC Standards, Milan, Italy) and was cultured in DMEM high glucose with stable L-glutamine, supplemented with 10% FBS, 100 U/mL penicillin, 100 µg/mL streptomycin (complete growth medium) with incubation at 37 °C under 5% CO_2_ atmosphere.

### 2.3. HMGCoAR Activity Assay

The experiments were carried out following the manufacturer’s instructions and optimized protocol [18]. More details are reported in the Supplementary Materials.

### 2.4. MTT Assay

A total of 3 × 10^4^ HepG_2_ cells/well were seeded in 96-well plates and treated with peptide H3 (0.00001, 0.0001, 0.001, 0.01, 0.1, and 1.0 mM) or vehicle (H_2_O) in complete growth media at 37 °C for 48 h under 5% CO_2_ atmosphere. MTT experiments were performed using the protocol reported in the Supplementary Materials.

### 2.5. In-Cell Western (ICW) Assay

For the experiments, a total of 3 × 10^4^ HepG2 cells/well were seeded in 96-well plates. The following day, the cells were washed with PBS and then starved overnight (O/N) in DMEM without FBS nor antibiotics. After starvation, HepG2 cells were treated with peptide H3 (25.0 µM) and vehicle (H_2_O) for 24 h at 37 °C under 5% CO_2_ atmosphere. The ICW assay was performed using the protocol reported in the Supplementary Materials.

### 2.6. Fluorescent LDL Uptake

HepG2 cells (3 × 10^4^/well) were seeded in 96-well plates and kept in complete growth medium for 2 days before treatment. On the third day, cells were washed with PBS and then starved overnight (O/N) in DMEM without FBS nor antibiotics, and experiments were performed using a protocol already optimized [18]. Further details are reported in the Supplementary Materials.

### 2.7. Western Blot Analysis

Immunoblotting experiments were performed using an optimized protocol [18]. Details are available in the Supplementary Materials.

### 2.8. Quantification through ELISA of PCSK9 Secreted by HepG2 Cells

The supernatants collected from treated HepG2 cells (25.0 μM H3) were centrifuged at 600× *g* for 10 min at 4 °C, and an ELISA assay was performed using a protocol already optimized [18]. Further details are reported in the Supplementary Materials.

### 2.9. Computational Methods

Docking simulations were performed by using the already-published computational procedure [19]. Briefly, the H3 peptide was built by using the VEGA program and its conformational profile was explored by quenched MonteCarlo analysis [20]. The minimized peptide was then docked within the previously prepared HMGCoAR structure. Docking simulations were carried out using PLANTS and the 10 generated poses ranked by the ChemPLP scoring function with the speed equal to 1 [21]. The so-obtained complexes were finally minimized and rescored, as implemented in ReScore+ [22].

### 2.10. Statistical Analysis

All the data sets were checked for normal distribution by the D’Agostino and Pearson test. Since they are all normally distributed with *p*-values < 0.05, we proceeded with statistical analyses by *t*-test and One-Way ANOVA followed by the Dunnett’s and Tukey’s post-hoc tests and using Graphpad Prism 9 (San Diego, CA, USA). Values were reported as means ± S.D.; *p*-values < 0.05 were considered to be significant.

## 3. Results

### 3.1. Peptide H3 Drops HMGCoAR Activity In Vitro

**Figure 1:**
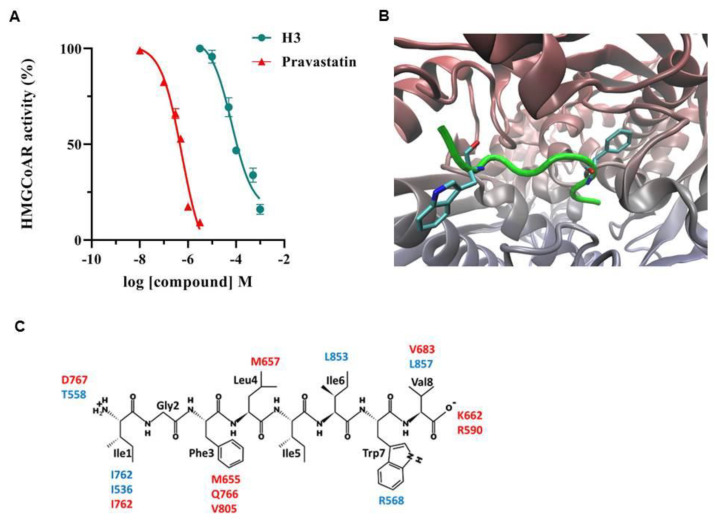
In vitro inhibition and in silico analysis of the peptide H3 interaction with HMGCoAR. In (**A**), points indicate the effects of H3 and pravastatin on the HMGCoAR activity. The data points represent the averages ± S.D. of three independent experiments in triplicate. (**B**) shows the putative pose of H3 (in green) within the binding cavity of HMGCoAR, which is placed at the interface between the two monomers (drawn in blue and red). (**C**) details the key interactions stabilized by each residue of H3 (the residue labels are colored according to the monomer).

HMGCoAR is the known target of statins, the main drugs used for the therapy of hypercholesterolemia [5,23]. Since the HP is able to drop the catalytic activity of HMGCoAR, we decided to evaluate the inhibitory ability of peptide H3 in vitro using the purified catalytic domain of HMGCoAR, testing concentrations ranging from 10 to 1000 μM. As shown in Figure 1A, the catalytic activity of HMGCoAR was inhibited by peptide H3 in a dose-dependent manner with an IC_50_ value of 59 μM. Pravastatin, which was used as positive control, instead inhibits enzyme activity with an IC_50_ equal to 0.55 µM. This value agrees with the IC_50_ of 0.47 µM reported in the literature [24]. Pravastatin is, thus, at least 100-fold more potent than H3 (Figure 1A).

To obtain further insight in the interaction of peptide H3 and HMGCoAR, a docking study was undertaken. Figure 1B reports the computed putative complex between H3 and HMGCoAR, revealing that peptide H3 is conveniently accommodated at the interface between the two monomers, with which it stabilizes a diverse pattern of interactions. Figure 1B also indicates that Phe-3 is nicely inserted within a narrow subpocket, mostly lined by hydrophobic residues. Similarly, Trp-7 approaches the Lα1 helix of the other monomer, where it stabilizes a clear charge-transfer interaction with Arg-568. Although both charged termini are involved (as expected) in key ion-pairs, Figure 1C emphasizes the remarkable role played by the hydrophobic contacts in the H3 binding. In detail, almost all the central residues are engaged in hydrophobic interactions involving both alkyl side chains and methionine residues, which can also elicit π-sulphur interactions with aromatic residues (i.e., Phe-5). A comparison with the key interactions stabilizing the resolved complex of HMGCoAR with statins [25] reveals an interesting agreement with those observed for the H3 peptide, which concern both the basic residues bridging the ligand’s carboxylate (K662 and R590) and the hydrophobic side chains which also approach the apolar moieties of the statins (V683, L853, L857).

### 3.2. Peptide H3 Effects on the Cell Vitality of HepG2

MTT experiments were performed to exclude any potential effect of peptide H3 on HepG2 cellular vitality. Because HepG2 cells are a slow growing cell line with an average doubling time of around 2 days, they were incubated for 48 h with different concentrations of peptide H3 before MTT assay. After a 48 h treatment, no effect on HepG2 viability was observed up to 100 µM versus the control cells (C). Instead, a reduced cell viability equal to 19.63 ± 0.59% was detected after the treatment with 1 mM H3 (Figure S1). These results are in line with previous evidences [15,17]. Based on these results, the following experiments, aimed at investigating the molecular and functional effects of peptide H3, were assessed at 25.0 µM, a dose which is 40-fold less concentrated than the lowest dose (1 mM) affecting cell viability.

### 3.3. Peptide H3 Modulates the LDLR Pathway

**Figure 2:**
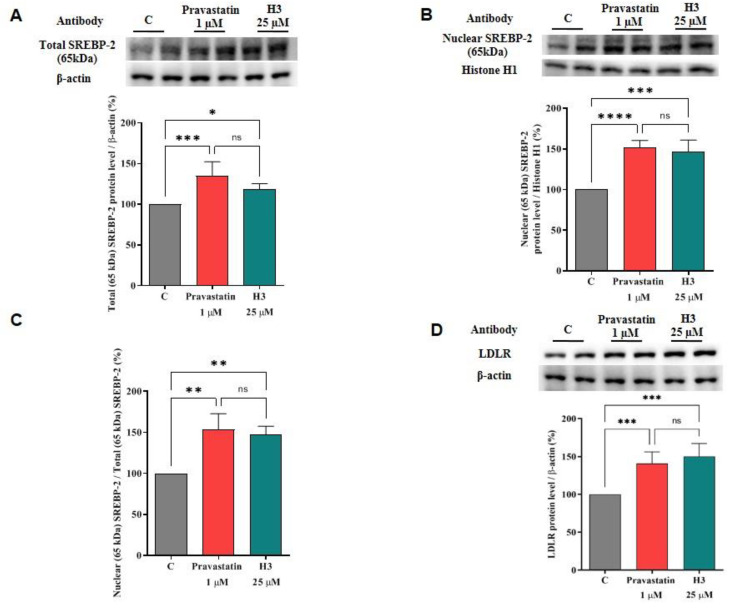
Effect of peptide H3 on LDLR pathway. HepG2 cells were treated with peptide H3 (25.0 μM) or pravastatin (1.0 µM). Mature SREBP-2 (65 KDa) on total lysate, mature SREBP-2 (65 KDa) on nuclear fraction of HepG2 cells, LDLR, β-actin, and histone H1 immunoblotting signals were detected using specific anti-SREBP-2, anti-LDLR, anti-β-actin, and anti-histone H1 primary antibodies, respectively. The total (**A**) and nuclear (**B**) SREBP-2 (65 KDa) signals, as well as the LDLR (**D**) signals, were quantified by ImageJ Software and normalized with β-actin or with histone H1 signals. Panel (**C**) indicates the nuclear/total SREBP-2 (65 KDa) ratio. Bars represent the averages of three independent experiments ± S.D., each performed in duplicate. ns: not significant, (*) *p* < 0.05, (**) *p* < 0.01, (***) *p* < 0.001, and (****) *p* < 0.0001 vs. control: C.

For assessing the ability of peptide H3 to modulate the LDLR pathway, immunoblotting experiments were performed on HepG2 cell lysates obtained after their treatment with peptide H3 at 25.0 μM. In parallel, HepG2 cells were also treated with pravastatin (1.0 µM) as the reference compound. The results suggest that the LDLR pathway was effectively activated after 24 h treatment (Figure 2). In more detail, peptide H3 induced an up-regulation of the total protein level of the mature SREBP-2 transcription factor (65 KDa) by 118.3% ± 7.00% versus the untreated cells (*p* < 0.05, Figure 2A). In addition, H3 increased the mature nuclear SREBP-2 protein levels (65 KDa) up to 147.0 ± 13.8% (*p* < 0.001, Figure 2B). In agreement with these data, an improvement of the nuclear/total mature SREBP-2 (65 KDa) ratio up to 147.1 ± 10.4% was detected in the HepG2 cells treated with the peptide compared to the untreated cells (*p* < 0.01, Figure 2C). In parallel, the precursor form of SREBP-2 (125 KDa) was also detected in the cytosolic fraction of HepG2 cells, and the ratio of mature (65 KDa)/precursor (125 KDa) SREBP-2 protein levels was calculated (Figure S2A,B). The results indicate that peptide H3 improved the precursor SREBP-2 (125 KDa) protein up to 157.5 ± 10.9% (*p* < 0.05) and the mature (65 KDa)/precursor (125 KDa) SREBP-2 protein level ratio up to 122.1 ± 1.2% (*p* < 0.01) versus the untreated cells (Figure S2A,B), confirming the improvement of mature SREBP-2 protein levels in the nuclear fraction, which, consequently, led to an increment of total LDLR proteins up to 150.2 ± 17.02% versus the control (*p* < 0.001, Figure 2D). Under the same conditions, pravastatin (1.0 µM) improved the protein levels of total mature SREBP-2 (65 KDa) and nuclear mature SREBP-2 (65 KDa) targets by 134.9 ± 17.3% (*p* < 0.001, Figure 2A) and 152.3 ± 8.2% (*p* < 0.0001, Figure 2B), respectively, versus the untreated cells. The ratio between the nuclear mature SREBP-2 (65 KDa) and total mature SREBP-2 (65 KDa) was increased up to 153.2 ± 19.5% (*p* < 0.01, Figure 2C). In addition, pravastatin increased the precursor SREBP-2 (125 KDa) protein levels in the cytoplasmic fraction up to 140.8 ± 9.0% (*p* < 0.05) leading to an augmentation of the mature (65 KDa)/precursor (125 KDa) SREBP-2 protein level ratio up to 122.4 ± 2.6% (*p* < 0.01) (Figure S2A,B). In agreement with the improved mature SREBP-2 levels in the nuclear fraction of HepG2 cells, pravastatin (1.0 µM) increased the LDLR up to 140.8 ± 15.35% (*p* < 0.001, Figure 2D) versus the control cells.

### 3.4. Peptide H3 Modulates Intracellular HMGCoAR Protein Levels

**Figure 3:**
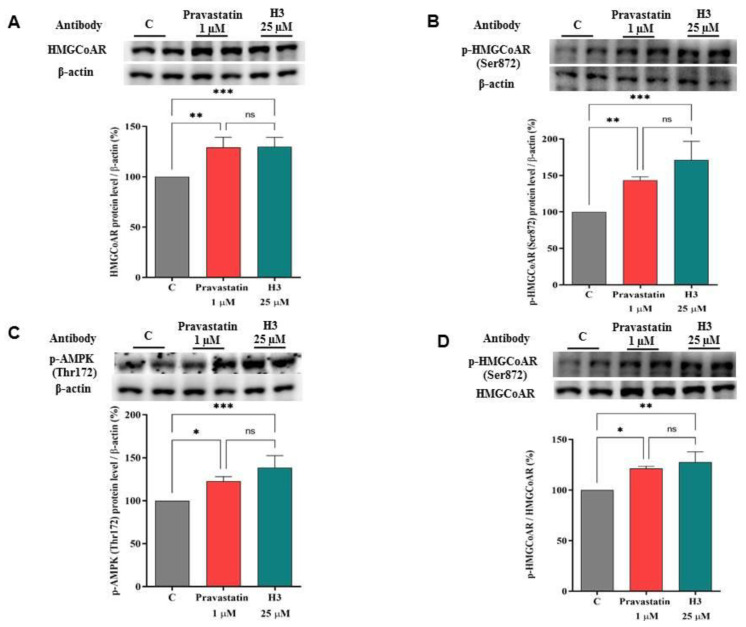
Peptide H3 and pravastatin increased HMGCoAR protein levels (**A**). Peptide H3 and pravastatin improved the inactive phosphorylated HMGCoAR (p-HMGCoAR) protein levels (**B**) due to the activation of AMPK through the augmentation of its phosphorylation on Thr172 residue (**C**). The ratio between p-HMGCoAR and total HMGCoAR was calculated after treatment with peptide H3 versus the C sample (**D**). Bars represent the averages of three independent experiments ± S.D., each performed in duplicate. ns: not significant, (*) *p* < 0.05, (**) *p* < 0.01, (***) *p* < 0.001 vs. control: C.

Because of the augmentation of the SREBP-2 transcription factor followed by an improvement of the LDLR protein levels, an increase of the HMGCoAR protein levels was also observed after 24 h treatment with peptide H3. The HMGCoAR protein levels were improved by 129.9 ± 9.16% (*p* < 0.001, Figure 3A) versus the control, while pravastatin (1.0 µM) increased the enzyme protein by 129.3 ± 9.33% (*p* < 0.01, Figure 3A). The literature evidences suggest that statins induce the activation of the AMPK-pathway through the augmentation of its phosphorylation on threonine 172 in different cellular systems [26,27,28], which in turn produces an inhibition of HMGCoAR activity through its phosphorylation on Ser872 residue [29], which is the phosphorylation site of AMPK. In light of this observation, and with the aim of comparing the hypocholesterolemic behavior of H3 with statin, further immunoblotting experiments were designed to investigate the effect of the treatment with peptide H3 and pravastatin on AMPK activation and HMGCoAR inactivation (AMPK substrate). Notably, the augmentation of phosphorylation levels of HMGCoAR (serine 872, AMPK phosphorylation site) were detected up to 171.2 ± 25.71% versus the control (*p* < 0.001, Figure 3B) upon cellular treatment with H3. This result is in line with the increase of AMPK phosphorylation (threonine 172) up to 138.5 ± 14.04% versus the control (*p* < 0.001, Figure 3C). In line with the literature evidences, as a reference compound, pravastatin (1.0 µM) was able to increase the AMPK phosphorylation (threonine 172) up to 122.7 ± 5.23% (*p* < 0.05, Figure 3C), followed by the increment of phosphorylation levels of HMGCoAR (serine 872) up to 143.5 ± 4.64% (*p* < 0.01, Figure 3B).

The p-HMGCoAR/total HMGCoAR ratio of the H3-treated, pravastatin-treated, and untreated cells were also calculated. The ratio of treated cells was higher than that of untreated ones. In fact, the peptide H3 and pravastatin increased it up to 127.6 ± 10.21% (*p* < 0.01, Figure 3D) and 121.3 ± 2.17% (*p* < 0.05, Figure 3D), respectively.

### 3.5. Peptide H3 Increases the Expression of LDLR Localized on Cellular Membranes and Modulates LDL-Uptake in the HepG2 Cell Environment

**Figure 4:**
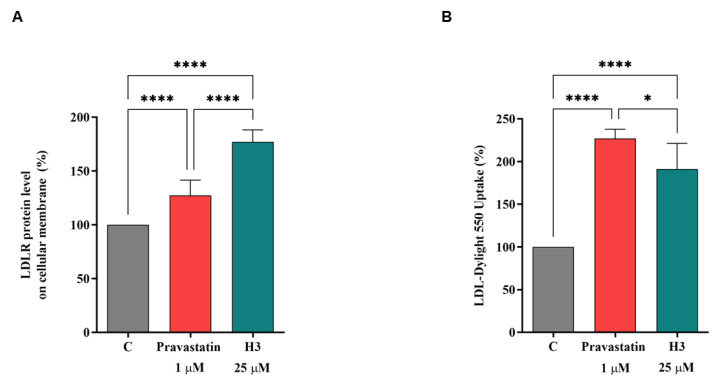
HepG2 cells were treated with peptide H3 (25.0 μM) or pravastatin (1.0 µM) for 24 h. (**A**) The percentage of LDLR protein up-regulation was measured by ICW. (**B**) The specific fluorescent LDL-uptake signals were analyzed by Synergy H1 (Biotek). The data points represent the averages ± S.D. of three experiments in triplicate. (*) *p* < 0.05, (****) *p* < 0.0001 vs. control: C.

The capacity of peptide H3 to modulate the LDLR protein levels on the hepatocyte cellular surface was investigated using an ICW assay, i.e., a quantitative colorimetric cell-based assay [30]. An improvement of the LDLR protein levels specifically localized on the cellular membrane of hepatocytes was observed up to 176.9 ± 11.31% (*p* < 0.0001, Figure 4A). As reference compound, pravastatin (1.0 µM) also increased the LDLR protein levels by 127.3 ± 14.31% (*p* < 0.0001, Figure 4A). From a functional point of view, the augmentation of the membrane LDLR protein levels led to the improved ability of HepG2 cells to absorb LDL from the extracellular environment by 191.1 ± 30.30% after the treatment with peptide H3 at the same concentration of 25.0 µM (*p* < 0.0001, Figure 4B). In the same conditions, pravastatin (1.0 µM) improved the capacity of HepG2 cells to absorb extracellular LDL by 226.9 ± 11.05% (*p* < 0.0001, Figure 4B). The statin increases the LDL uptake more efficiently than the peptide, whereas H3 is more efficient in increasing the LDLR levels. This apparent contradictory fact may be explained by considering that the LDL-uptake assay and ICW display different sensitivities. Indeed, the former (LDL-uptake) works in fluorescence, which is a more sensitive assay than the latter (ICW), which is a colorimetric test. However, these results indicate that the hypocholesterolemic behavior of peptide H3 is similar to that of pravastatin.

### 3.6. The Effect of Peptide H3 on the Modulation of PCSK9 Protein and Release Levels

**Figure 5:**
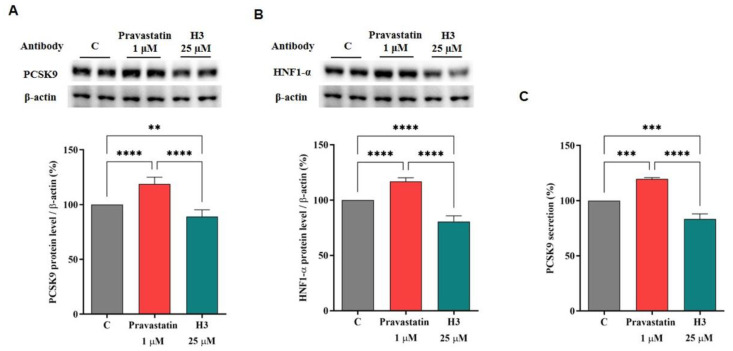
After 24 h treatment with peptide H3 (25.0 μM) or pravastatin (1.0 µM), effects on the PCSK9 protein levels (**A**), effects on the HNF1-α protein levels (**B**), and effects on mature PCSK9 release (**C**). The Panel signals were quantified by ImageJ Software and normalized with β-actin signals. Data points represent the averages ± S.D. of three experiments in duplicate. (**) *p* < 0.001, (***) *p* < 0.001, and (****) *p* < 0.0001 vs. control: C.

Previous studies have evidenced a similar hypocholesterolemic effect of hempseed hydrolysates (HP) to statins, leading to the increase of the PCSK9 protein levels [12]. To assess if peptide H3 can perform a satisfactory modulation on the hepatic intracellular PCSK9 protein and release levels or maintain the same ability of HP for PCSK9, dedicated experiments were conducted. Hence, HepG2 cells were treated with peptide H3 at 25.0 µM to investigate the modulation on PCSK9 and its transcription factor HNF1-α. Surprisingly, the results clearly indicate that peptide H3 caused a 10.85 ± 6.12% reduction of intracellular PCSK9 protein levels versus the control (*p* < 0.01, Figure 5A), playing an opposite effect on PCSK9 compared with HP. As expected, this finding agrees with the ability of peptide H3 to reduce the HNF1-α transcription factor levels by 19.40 ± 5.18% compared to the untreated cells (*p* < 0.0001, Figure 5B). In contrast, after treatment with pravastatin (1.0 µM), the direct activation of HNF1-α was up to 116.9 ± 3.21% (*p* < 0.0001, Figure 5B), resulting in the increase of PCSK9 protein levels up to 118.8 ± 6.11% (*p* < 0.0001 Figure 5A).

In light of these results, the effect of peptide H3 on the regulation of the mature PCSK9 release was evaluated by ELISA assay. In agreement with the molecular results, peptide H3 could decrease the secretion of the mature PCSK9 by 16.58 ± 4.64% versus control (*p* < 0.001, Figure 5C), while pravastatin (1.0 µM) improved the release of PCSK9 up to 119.6 ± 1.18% (*p* < 0.001, Figure 5C).

## 4. Discussion

CVD is a multifactorial pathology in which oxidative and inflammatory status as well as hypercholesterolemia are among the main risk factors [31]. Peptide H3 is a multifunctional octapeptide, obtained from the hydrolysis of hempseed proteins using pepsin [15], that is transported intact by differentiated Caco-2 and is able to exert both antioxidant and anti-inflammatory activity in HepG2 cells [15,17]. These results prompted us to better investigate its multifunctional behavior. To achieve this goal, a deeper study was carried out for characterizing the potential effect of peptide H3 on cholesterol metabolism. Peptide H3 reduces the in vitro HMGCoAR activity with a dose-response trend and an IC_50_ value of 59 μM (Figure 1). Its activity is similar to peptide LTFPGSAED (from lupin protein hydrolysis with pepsin) and peptide VGVL (from amaranth protein hydrolysis with pepsin), displaying IC_50_ values equal to 68.4 and 50 µM, respectively [32]. On the contrary, peptide H3 is about 2.5-fold more potent than LILPKHSDAD (from lupin protein hydrolysis with pepsin) [18]. In addition, in contrast to other soybean peptides such as IAVPTGVA, IAVPGEVA, and LPYP (from soybean glycinin hydrolysis with pepsin), and YVVNPDNDEN and YVVNPDNNEN (from soybean β-conglycinin)—which are less effective inhibitors of HMGCoAR (IC_50_ values equal to 247, 222, 300 μM [33] and 150, and 200 μM [34], respectively)—peptide H3 exerts a more potent inhibitory activity of this enzyme (up to five-fold higher).

From a computational point of view, the docking results emphasize that a convenient HMGCoAR binding can be pursued by properly combining polar and hydrophobic interactions. This result is in agreement with the resolved HMGCoAR structures in complex with statins, and emphasizes that peptides which are too polar have to pay the unfavorable price of desolvation energy, regardless of their interacting capacity. In light of these evidences, the effect of peptide H3 on cholesterol metabolism was analyzed using human hepatic HepG2 cells, the major cell involved in plasma LDL cholesterol clearance due to its ability to express the highest number of active LDLR on its surface [35]. Indeed, HepG2 cells are globally considered a reliable model for investigating the cholesterol-lowering effects of bioactive agents from different sources [36,37,38,39,40].

Preliminary MTT experiments were carried out to exclude any potential dose impacting on cell viability. In line with previous evidences [15,17], the results confirmed that H3 is safe for HepG2 cells up to 100 µM (Figure S1). Based on these results, HepG2 cells were treated with 25 µM of peptide H3. Similarly to pravastatin (1 µM, the positive control), by inhibiting HMGCoAR activity, peptide H3 modulates the intracellular cholesterol pathway, leading to an increase of the LDLR and HMGCoAR protein levels through the modulation of SREBP-2 (Figure 2, Figure 3 and Figure S2). Notably, HMGCoAR is a highly-regulated enzyme [3]: it can be regulated long-term by the control of its synthesis and its degradation or short-term through phosphorylation or dephosphorylation [41]. In particular, its regulation is achieved through phosphorylation of Ser872 by AMPK, which decreases the enzyme activity [42].

The literature reports that some natural compounds such as policosanols are able to increase the phosphorylation of AMPK with the direct inhibition of HMGCoAR [43]. Furthermore, it is also known that statins are able to activate AMPK [44] with the consequence of a synergistic inhibition of HMGCoAR activity, its direct target [29]. In agreement with these evidences, our results provide a clear indication that, similarly to statins [26,27,28], peptide H3 increases the phosphorylation level of AMPK at the Thr172 residue of the catalytic subunit, which in turn produces an inhibition of HMGCoAR activity through its phosphorylation on the Ser872 residue [29], which is the phosphorylation site of AMPK (Figure 3). In line with these findings, an improvement of the p-HMGCoAR/HMGCoAR protein ratio with the consequence of a diminished enzyme activity was observed, in agreement with the literature [45] (Figure 3). Other plant peptides share the same mechanism of HMGCoAR inhibition, in particular IAVPGEVA, IAVPTGVA, and LPYP, deriving from soybean protein [38], and LTFPGSAED and LILPHKSDAD from lupin proteins [18]. In addition, it was demonstrated that this hempseed peptide increases the LDLR localized on HepG2 cell surfaces, leading from a functional point of view to the improved ability of these cells to clear extracellular LDL with a final cholesterol-lowering consequence (Figure 4). The cholesterol-lowering behavior of peptide H3 is similar to that of pravastatin; however, a different effect was observed on the PCSK9 protein levels. Interestingly, both PCSK9 and LDLR are co-regulated by SREBP-2 transcription factor [46,47]. However, since the HNF1-α binding site is unique to the PCSK9 promoter, and is not present in the LDLR promoter, the modulation of the PCSK9 transcription through HNF1-α does not affect the LDLR pathway. Thus, the co-regulation of PCSK9 from LDLR and other SREBP target genes is disconnected by the HNF1-α binding site [47,48]. In this context, it is known that statins increase the protein levels of PCSK9, which quench any effective LDL clearance by promoting LDLR degradation [49], thereby counteracting the therapeutic effects of these drugs [46].

Indeed, our results confirm that pravastatin improves the mature PCSK9 protein levels through HNF1-α enhancement; on the contrary, peptide H3 reduces both HNF1-α and PCSK9 levels. In line with these results, it was observed that only peptide H3 reduces the PCSK9 release in extracellular environments, whereas pravastatin was ineffective. This result contributes to explaining the better ability of peptide H3 to raise the level of the LDLR population localized on the surface of HepG2 cells than pravastatin. Indeed, the effect of peptide H3 on the PCSK9 protein and release levels is similar to lupin hydrolysate [38,39] but different from the behavior of the hempseed hydrolysates [12]. Indeed, only lupin hydrolysate is able to modulate the HMGCoAR activity and PCSK9 protein and release levels, whereas hempseed seems to exert the cholesterol lowering activity targeting HMGCoAR activity similarly to statin, without producing an effect on PCSK9 secretion and protein levels [12].

## 5. Conclusions

In this study, peptide H3 obtained from the hydrolysis of hempseed protein was shown to exert cholesterol-lowering effects, displaying a multifunctional activity which is better compared with hempseed hydrolysates (HP), especially in the modulation of PCSK9 protein and release levels. Since there is a lack of studies showing how peptide H3 affects PCSK9, relevant experiments will be carried out in the future to elucidate the specific mechanism. Indeed, it may be feasibly concluded that peptide H3 might be exploited in the future for the development of new dietary supplements and/or used as a scaffold for the synthesis of new peptidomimetics for the prevention of CVD and/or metabolic syndrome. In this context, it will be imperative to carry out in vivo experiments on suitable animal models to confirm H3 pleiotropic activity and to establish the right amount of peptides that should be consumed in the human diet or in supplements to achieve its health-promoting activity.

## Data Availability

Not applicable.

## Supplementary Materials

The following supporting information can be downloaded at: https://www.mdpi.com/article/10.3390/nu14091804/s1, Figure S1: effect of H3 on HepG2 viability; Figure S2: (A) Precursor SREBP-2 protein levels (125 KDa) in cytoplasmatic fraction; (B) precursor/mature SREBP-2 ratio.
