# Supplementary material for: Hempseed (Cannabis sativa) Peptide H3 (IGFLIIWV) Exerts Cholesterol-Lowering Effects in Human Hepatic Cell Line
Source: Nutrients. 2022 Apr 26;14(9):1804. doi: 10.3390/nu14091804 (PMC9101684; doi:10.3390/nu14091804)
Supplement: Supplementary file 1 [file nutrients-14-01804-s001.zip › nutrients-1675380-supplementary.pdf]

Supplementary Material

Hempseed (*Cannabis sativa*) Peptide H3 (IGFLIIWV) Exerts Cholesterol-Lowering Effects in Human Hepatic Cell Line

Jianqiang Li, Carlotta Bollati, Martina Bartolomei, Angelica Mazzolari, Anna Arnoldi, Giulio Vistoli and Carmen Lammi *

Department of Pharmaceutical Sciences, University of Milan, Via Mangiagalli 25, 20133 Milan, Italy; jianqiang.li@unimi.it (J.L.); carlotta.bollati@unimi.it (C.B.); martina.bartolomei@unimi.it (M.B.); angelica.mazzolari@unimi.it (A.M.); anna.arnoldi@unimi.it (A.A.); giulio.vistoli@unimi.it (G.V.)

* Correspondence: carmen.lammi@unimi.it; Tel.: +39-02-5031-9372

Citation: Li, J.; Bollati, C.; Bartolomei, M.; Mazzolari, A.; Arnoldi, A.; Vistoli , G. Hempseed (*Cannabis sativa*) Peptide H3 (IGFLIIWV) Exerts Cholesterol-Lowering Effects in Human Hepatic Cell Line. *Nutrients* **2022**, *14*, 1804. https://doi.org/10.3390/nu14091804

Academic Editor: Satoshi Nagaoka

Received: 25 March 2022
Accepted: 24 April 2022
Published: 26 April 2022

Publisher's Note: MDPI stays neutral with regard to jurisdictional claims in published maps and institutional affiliations.

Copyright: © 2022 by the authors. Submitted for possible open access publication under the terms and conditions of the Creative Commons Attribution (CC BY) license (https://creativecommons.org/licenses/by/4.0/).

2. Materials and Methods

2.1. Chemicals

The Dulbecco's modified Eagle's medium (DMEM), stable L-glutamine, fetal bovine serum (FBS), phosphate buffered saline (PBS), penicillin/streptomycin, chemiluminescent reagent, and 96-well plates were purchased from Euroclone (Milan, Italy). The HMGCoAR assay kit, bovine serum albumin (BSA), Janus Green B, formaldehyde, Ethylenediaminetetraacetic acid (EDTA), HEPES, Glycerol, Nonidet P40 Substitute (NP-40), EDTA-free Protease Inhibitor Cocktail (Roche), NaCl, $MgCl_2$, HCl and H_2SO_4 were from Sigma-Aldrich (St. Louis, MO, USA). The antibody against LDLR and the 3, 3',5, 5'-tetramethylbenzidine (TMB) substrate were bought from Thermo Fisher Scientific (Waltham, MA, USA). The Quantikine ELISA kit was bought from R&D Systems (Minnneapolis, MN, USA). The LDL-DyLight™ 550 was from Cayman Chemical (Ann Arbor, MI, USA). The CircuLex PCSK9 *in vitro* binding Assay Kit was from CircuLex (CycLex Co., Nagano, Japan). The peptides H3 was synthesized by the company GeneScript (Piscataway, NJ, USA) at > 95% purity. The antibody against HMGCoAR was bought from Abcam (Cambridge, UK). Phenylmethanesulfonyl fluoride (PMSF), Na-orthovanadate inhibitors, and the antibodies against rabbit Ig-horseradish peroxidase (HRP), mouse Ig-HRP, rabbit Histone H1, and SREBP-2 (which recognizes epitope located in a N-terminal region between 833–1141, in common with both mature (65 KDa) and precursor (125 KDa) forms) were purchased from Santa Cruz Biotechnology Inc. (Santa Cruz, CA, USA). The inhibitor cocktail Complete Midi was from Roche (Basel, Switzerland). Mini protean TGX pre-cast gel 7.5% and Mini nitrocellulose Transfer Packs were purchased from BioRad (Hercules, CA, USA).

2.2. HepG2 Cell Culture Conditions and Treatment

The HepG2 cell line was bought from ATCC (HB-8065, ATCC from LGC Standards, Milan, Italy) and was cultured in DMEM high glucose with stable L-glutamine, supplemented with 10% FBS, 100 U/mL penicillin, 100 μg/mL streptomycin (complete growth medium) with incubation at 37 °C under 5% CO_2 atmosphere.

2.3. HMGCoAR Aactivity Assay

The experiments were carried out following the manufacturer instructions and optimized protocol [18]. The assay buffer, NADPH, substrate solution, and

HMGCoAR were provided in the HMGCoAR Assay Kit (Sigma Aldrich SRL, Milan, Italy). The experiments were carried out following the manufacturer instructions at 37 °C. In particular, each reaction (200 μL) was prepared adding the reagents in the following order: 1 X assay buffer, a 10–1000 μM doses of H3 or vehicle (C), the NADPH (4 μL), the substrate solution (12 μL), and finally the HMGCoAR (catalytic domain) (2 μL). Subsequently, the samples were mixed and the absorbance at 340 nm read by the microplate reader Synergy H1 (Winooski, VT, USA) at time 0 and 10 min. The HMGCoAR-dependent oxidation of NADPH and the inhibition properties of peptides were measured by absorbance reduction, which is directly proportional to enzyme activity.

2.4. MTT Assay

A total of 3×10^4 $HepG_2$ cells/well were seeded in 96-well plates and treated with 0.00001, 0.0001, 0.001, 0.01, 0.1 and 1.0 mM of peptide H3, respectively, or vehicle (H_2O) in complete growth media for at 37 °C under 5% CO_2 atmosphere for 48 h. Subsequently, the treatment solvent was aspirated, and 100 μL/well of 3-(4,5 dimethylthiazol-2-yl)-2,5-diphenyltetrazolium bromide (MTT) filtered solution was added. After 2 h of incubation at 37 °C under 5% CO_2 atmosphere, the 0.5 mg/mL solution was aspirated, and 100 μL/well of the lysis buffer (8 mM HCl + 0.5% NP-40 in DMSO) was added. After 5 min of slow shaking, the absorbance at 575 nm was read on the Synergy H1 fluorescence plate reader (Biotek, Bad Friedrichshall, Germany).

2.5. In-Cell Western (ICW) Assay

For the experiments, a total of 3 X 10^4 HepG2 cells/well were seeded in 96-well plates. The following day, cells were washed with PBS and then starved overnight (O/N) in DMEM without FBS and antibiotics. After starvation, HepG2 cells were treated with peptide H3 (25.0 μM) and vehicle (H_2O) for 24 h at 37 °C under 5% CO_2 atmosphere. Subsequently, they were fixed in 4% paraformaldehyde for 20 min at room temperature (RT). Cells were washed 5 times with 100 μL of PBS/well (each wash was for 5 min at RT) and the endogenous peroxides activity quenched adding 3% H_2O_2 for 20 min at RT. Non-specific sites were blocked with 100 μL/well of 5% bovine serum albumin (BSA, Sigma) in PBS for 1.5 h at RT. LDLR primary antibody solution (1:3000 in 5% BSA in PBS, 25 μL/well) was incubated overnight (O/N) at + 4 °C. Subsequently, the primary antibody solution was discarded and each sample was washed 5 times with 100 μL/well of PBS. Goat anti-rabbit Ig-HRP secondary antibody solution (Santa Cruz) (1:6000 in 5% BSA in PBS, 50 μL/well), was added and incubated 1 h at RT. The secondary antibody solution was washed 5 times with 100 μL/well of PBS. Freshly prepared TMB substrate (Pierce, 100 μL/well) was added and the plate was incubated at RT until desired color was developed. The reaction was stopped with 2 M H_2SO_4 and then the absorbance at 450 nm was measured using the microplate reader Synergy H1 (Winooski, VT, USA). After the read, cells were stained by adding 1 X Janus Green stain, incubating for 5 min at RT. The dye was removed and the sample washed 5 times with water. Afterward 100 μL 0.5 M HCl for well were added and incubated for 10 min. After 10 min shaking, the OD at 595 nm was measured using the microplate reader Synergy H1 (Winooski, VT, USA).

2.6. Fluorescent LDL Uptake

HepG2 cells (3 X 10^4/well) were seeded in 96-well plates and kept in complete growth medium for 2 days before treatment. The third day, cells were washed with PBS and then starved overnight (O/N) in DMEM without FBS and antibiotics. After starvation, they were treated with peptide H3 (25.0 μM), and vehicle (H_2O) for 24 h with at 37 °C under 5% CO_2 atmosphere. At the end of the

treatment, the culture medium was replaced with 50 μL/well LDL-DyLight™ 550 working solution (Cayman Chemical Company, Ann Arbor, MI, USA) prepared in DMEM without FBS and antibiotics. The cells were additionally incubated for 2 h at 37 °C and then the culture medium was aspirated and replaced with PBS (100 μL/well). The degree of LDL uptake was measured using the Synergy H1 fluorescent plate reader (Winooski, VT, USA) (excitation and emission wavelengths 540 and 570 nm, respectively). Fluorescent LDL-uptake was finally assessed following optimized protocol [19].

2.7. Western Blot Analysis

Immunoblotting experiments were performed using optimized protocol [18]. A total of 1.5 X 10^5 HepG2 cells/well (24-well plate) were treated with 25.0 μM of H3 for 24 h. After each treatment, the supernatants were collected and stored at -20 °C; cells were scraped in 40 μL ice-cold lysis buffer (RIPA buffer + inhibitor cocktail + 1:100 PMSF + 1:100 Na-orthovanadate + 1:1000 β-mercaptoethanol) and transferred in ice-cold microcentrifuge tubes. After centrifugation at 13,300 g for 15 min at 4 °C, the supernatants were recovered and transferred into new ice-cold tubes. For the mature SREBP-2 nuclear isolation, a total of 7.5 × 10^5 HepG2 cells/well (6-well plates) were treated with 25.0 μM of H3, pravastatin 1.0 μM or vehicle (H_2O) in complete growth media for at 37 °C under 5% CO_2 atmosphere for 24h. Therefore, cells were collected with PBS ice-cold and centrifuged 1500 rmp for 5 min at 4°C. The pellet was incubated with 20 μL of Nuclei Isolation Buffer A (10 mM HEPES pH 7.9, 1.5 mM MgCl2, 10 mM KCl, 0.2 mM EDTA, 0.1% β-mercaptoethanol, 0.5 mM PMSF, 0.1 % NP-40, one cOmplete™ Mini, EDTA-free Protease Inhibitor Cocktail) on ice for 10 min and then centrifuged 12000 rpm for 10 min at 4°C. Subsequently, the pellet was incubated with 20 μL of Nuclei Isolation Buffer B (20 mM HEPES pH 7.9, 1.5 mM MgCl2, 25% Glycerol, 0.2 mM EDTA, 0.1% β-mercaptoethanol, 0.5 mM PMSF, 0.42 M NaCl, one cOmplete™ Mini, EDTA-free Protease Inhibitor Cocktail) on ice for 20 min and then centrifuged 12000 rpm for 10 min at 4°C and the supernatant was collected (nucleus isolate). Total proteins were quantified by the Bradford's method and 50 μg of total proteins loaded on a pre-cast 7.5% Sodium Dodecyl Sulfate-Polyacrylamide (SDS-PAGE) gel at 130 V for 45 min. Subsequently, the gel was pre-equilibrated in H_2O for 5 min at room temperature (RT) and transferred to a nitrocellulose membrane (Mini nitrocellulose Transfer Packs,) using a Trans-Blot Turbo at 1.3 A, 25 V for 7 min. Target proteins, on milk or BSA blocked membrane, were detected by primary antibodies as follows: anti-SREBP-2, anti-LDLR, anti-HMGCoAR, anti-PCSK9, anti HNF1-α, anti-p-HMGCoAR, anti-p-AMPK and anti-β-actin. Secondary antibodies conjugated with HRP and a chemiluminescent reagent were used to visualize target proteins and their signal was quantified using the Image Lab Software (Biorad, Hercules, CA, USA). The internal control β-actin was used to normalize loading variations.

2.8. Quantification of PCSK9 Secreted by HepG2 Cells through ELISA

The supernatants collected from treated HepG2 cells (25.0 μM of H3) were centrifuged at 600 X g for 10 min at 4 °C and ELISA assay performed using protocol already optimized [18]. They were recovered and diluted to the ratio 1:10 with DMEM in a new ice-cold tube. PCSK9 was quantified by ELISA (R&D System, Minneapolis, MN, USA). Briefly, the experiments were carried out at 37 °C, following the manufacturer's instructions. Before starting the assay, human PCSK9 standard curve (20.0, 10.0, 5.0, 2.5, 1.25, and 0.625 ng/mL) was prepared by serial dilutions from a 40 ng/mL stock. 100 μL of the Assay Diluent RD1-9 (provided into the kit) were placed in each well, before adding the standards and the samples (50 μL) and incubating the ELISA plate for 2 h at RT. Subsequently, wells were washed 4 times with the wash buffer, and 200 μL of

human PCSK9-conjugate (HRP-labelled anti-PCSK9) was added to each well for 2 h at RT. Following aspiration, wells was washed 4 times with the kit wash buffer. After the last wash, 200 µL of substrate solution were added to the wells and allowed to incubate for 30 min at RT. The reaction was stopped with 50 µL of the stop solution (2 M sulfuric acid) and the absorbance at 450 nm was measured using Synergy H1 microplate (Winooski, VT, USA).

2.9. Computational Methods.

Docking simulations were performed by using the already published computational procedure. [26] Briefly, the H3 petide was built by using the VEGA program and its conformational profile was explored by quenched MonteCarlo analysis [20]. The minimized peptide was then docked within the previously prepared HMGCoAR structure. Docking simulation were carried out using PLANTS and generated 10 poses ranked by the ChemPLP scoring function with the speed equal to 1 [21]. The so obtained complexes were finally minimized and rescored as implemented in ReScore+ [22].

2.10. Statistical Analysis

All the data sets were checked for normal distribution by D'Agostino and Pearson test. Since they are all normally disturbed with *p*-values < 0.05, we proceeded with statistical analyses by T-Test and One-Way ANOVA followed by Dunnett's and Tukey's post-hoc tests and using Graphpad Prism 9 (San Diego, CA, USA). Values were reported as means ± S.D.: *p*-values < 0.05 were considered to be significant.

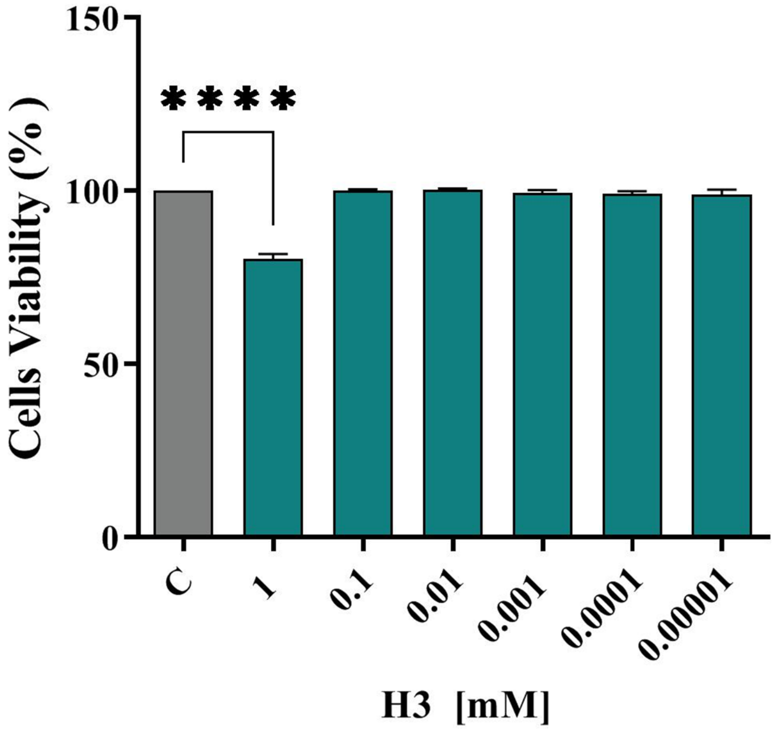

Figure S1. Effect of H3 on HepG2 cell viability. The bar graphs indicating the results of cell viability assay of HepG2 cells after peptide H3 (0.00001 – 1 mM) treatment for 48 h. The data points represent the averages ± S.D. of three experiments in triplicate. (****) p < 0.0001 vs control (C).

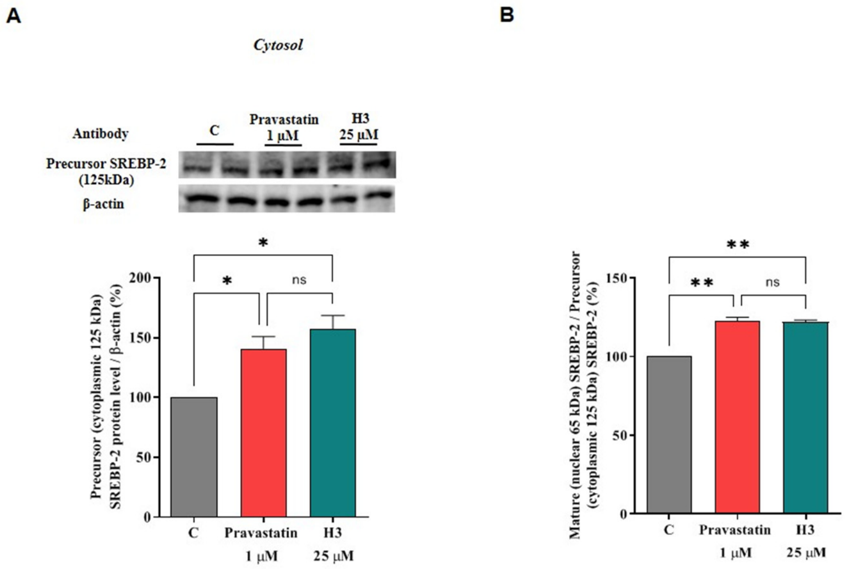

Figure S2. Effect of peptide H3 and pravastatin on the modulation of precursor SREBP-2 levels in cytoplasmatic fraction (A) and mature (nuclear 65 KDa)/precursor (125 KDa) SREBP-2 ratio (B). C: control; (* p< 0.5), (** p<0.01).
